# Identification of circRNA and mRNA expression profiles and functional networks of vascular tissue in lipopolysaccharide‐induced sepsis

**DOI:** 10.1111/jcmm.15424

**Published:** 2020-05-25

**Authors:** Mu‐Wen Nie, Ye‐Chen Han, Zhu‐Jun Shen, Hong‐Zhi Xie

**Affiliations:** ^1^ Department of Cardiology Chinese Academy of Medical Sciences and Peking Union Medical College Peking Union Medical College Hospital Beijing China

**Keywords:** aorta, circular RNAs, microarray, sepsis

## Abstract

Sepsis is the most common cause of death in intensive care units. This study investigated the circular RNA (circRNA) and mRNA expression profiles and functional networks of the aortic tissue in sepsis. We established a lipopolysaccharide (LPS)‐induced rat sepsis model. High‐throughput sequencing was performed on the aorta tissue to identify differentially expressed (DE) circRNAs and mRNAs, which were validated by real‐time quantitative polymerase chain reaction (RT‐qPCR). Bioinformatic analysis was carried out and coding and non‐coding co‐expression (CNC) and competing endogenous RNA (ceRNA) regulatory networks were constructed to investigate the mechanisms. In total, 373 up‐regulated and 428 down‐regulated circRNAs and 2063 up‐regulated and 2903 down‐regulated mRNAs were identified. Gene Ontology (GO) and Kyoto Encyclopedia of Genes and Genomes (KEGG) analyses of mRNAs showed that the down‐regulated genes were mainly enriched in the process of energy generation. CNC and ceRNA regulatory networks were constructed with seven DE circRNAs. The results of functional enrichment analysis of CNC target genes revealed the important role of circRNAs in inflammatory response. The ceRNA network also highlighted the significant enrichment in calcium signalling pathway. Significant alterations in circRNAs and mRNAs were observed in the aortic tissue of septic rats. In addition, CNC and ceRNA networks were established.

## 1 INTRODUCTION

Sepsis is characterized with systemic inflammation, accelerated activity of the clotting cascade and excessive endothelial permeability.^1^ Sepsis is a life‐threatening disease that usually leads to multiple organ dysfunctions. It is a leading cause of death among patients in intensive care units. While there has been significant progress in the development of therapeutic interventions to reduce mortality in the past few years, the heavy mortality burden and healthcare costs associated with sepsis highlight the urgent need to better understand this devastating clinical syndrome and identify new targets for prevention and therapy. However, the underlying mechanism of sepsis is unclear.

Non‐coding RNAs (ncRNAs) are all functional RNAs that are not translated into proteins. The development of sequencing technology has revealed several new ncRNAs, of which circular RNA (circRNA) is known as a highly stable and conserved form of RNA mainly observed in cell cytoplasm.^2^, ^3^ Given the absence of the 5′‐3′ polar structure, circRNAs have interconnected 3′ and 5′ ends that form a covalently closed continuous loop. CircRNAs bind to and decrease the expression of their target miRNAs, thereby acting as sponges to inhibit transcription. This is how these molecules affect various pathophysiological processes in osteoarthritis, Alzheimer's disease and cancers.^4^, ^5^, ^6^, ^7^ Li et al^8^ found that the expression of circRNA‐ITCH is decreased in oesophageal cancer as compared with that in the surrounding tissue, suggestive of its inhibitory effect on oesophageal cancer. Although circRNAs have been related to sepsis‐induced myocardial depression and pulmonary injury, whether these molecules play key roles in lipopolysaccharide (LPS)‐induced vascular damage is yet unclear. The expression profile of circRNAs in sepsis‐induced vascular injury has not been reported.

Lipopolysaccharide, a component of the cell wall of gram‐negative bacteria, is known to cause endotoxic shock, disseminated intravascular coagulation and multiple organ failure.^9^ LPS is a single component of the pathogen‐associated molecular patterns released by gram‐negative organisms. Although the model established with LPS injection may not exactly reproduce the pathological features of bacterial infections and polymicrobial sepsis, sepsis and endotoxaemia are systemic inflammatory responses to infection and may even lead to tissue damage and multiple organ failure.^10^ Therefore, Wistar rats were intraperitoneally injected with LPS to establish an animal model of sepsis. Several septic patients have circulatory failure that leads to organ perfusion insufficiency and, eventually, organ failure. Although mean arterial pressure (MAP) is usually regarded as the organ perfusion pressure, human organs spend more time being perfused in the diastolic period than in the systolic period. Therefore, diastolic pressure is more important to maintain organ perfusion. Considering its wall thickness, dilation and elasticity, the aorta is very important for maintaining diastolic pressure. In this direction, we focused on the mechanism underlying aortic injury in sepsis to find any potential clinical significance for improving organ perfusion.

In this study, we performed high‐throughput sequencing of circRNAs and mRNAs in aorta tissues from sepsis rat model. The differentially expressed (DE) circRNAs and mRNAs were screened and identified. The potential coding and non‐coding co‐expression (CNC) and circRNA‐micro RNA (miRNA)‐mRNA competing endogenous RNA (ceRNA) networks were predicted, and significantly enriched pathways were analysed. The identification of these DE circRNAs and mRNAs may provide an insight into therapeutic targets and new diagnostic methods for future.

## 2 MATERIALS AND METHODS

### 2.1 Animals

Animal experiments were approved by the relevant national laws on the protection of animals. Male Wistar rats (230‐250 g, 6‐7 weeks old) were obtained from Charles River Laboratories (Beijing, China). All rats were housed at room temperature and a relative humidity of ~60% with a 12 hous light/dark cycle. Animals were fed with normal chow and water. Following acclimatization for 1 week, rats were randomly assigned into two groups as follows: LPS group (n = 5) and control group (n = 5). LPS rats were intraperitoneally injected with LPS (10 mg/kg, 5 mg LPS dissolved in 1 mL 0.9% saline).^11^ LPS was purchased from Sigma‐Aldrich Chemical Co. (L‐2880). After 24 hours of LPS injection, MAP was non‐invasively measured. Septic shock model was established when MAP decreased to 25%‐30% of the baseline value.^11^ The aortic tissue was isolated, immediately frozen in liquid nitrogen and stored at −80°C until analysis.

### 2.2 RNA extraction and circRNA and mRNA microarray

Total RNA from the aorta tissue was quantified using the NanoDrop ND‐1000. Sample preparation and microarray hybridization were performed as per the Arraystar standard protocols. Briefly, total RNA was digested using RNase R (Epicentre, Inc.) to remove linear RNAs and enrich circRNAs, which were then amplified and transcribed into fluorescent complementary RNA (cRNA) utilizing a random priming method (Arraystar Super RNA Labeling Kit; Arraystar). The labelled cRNAs were hybridized onto the Arraystar Rat circRNA Array (8 × 15K; Arraystar). After washing the slides, the arrays were scanned using the Agilent Scanner G2505C. The mRNA microarray data were also obtained using the Agilent Rat 4 × 44K Gene Expression Array.

Agilent Feature Extraction software (version 11.0.1.1) was used to analyse acquired array images. Quantile normalization and subsequent data processing were performed with the R software limma package. DE circRNAs or mRNAs between two groups were identified through volcano plot filtering and those between two samples were evaluated through fold‐change filtering. Hierarchical clustering was performed to reveal the distinguishable circRNA or mRNA expression patterns among samples.

### 2.3 Bioinformatic analyses

Gene Ontology (GO) and Kyoto Encyclopedia of Genes and Genomes (KEGG) pathway analyses were carried out using standard techniques. GO enrichment analysis was based on three aspects, namely, biological process (BP), cellular component (CC) and molecular function (MF), and carried out to assess the functional roles of the top 10 significantly enriched target genes. KEGG pathway enrichment revealed the signalling networks of DE circRNAs associated with sepsis.

### 2.4 Validation of candidate circRNAs and mRNAs using real‐time quantitative polymerase chain reaction (RT‐qPCR)

We performed RT‐qPCR to verify the results of the microarray analysis for 20 DE circRNAs and 20 DE mRNAs. In brief, the total RNA extracted from the aorta tissue samples of two groups (n = 5 for each group) was reverse‐transcribed into complementary DNA (cDNA) using SuperScript™ III Reverse Transcriptase (Invitrogen). RT‐qPCR Master Mix (Arraystar, Inc.) and ViiA 7 Real‐Time PCR system (Applied Biosystems; Thermo Fisher Scientific, Inc.) were used for RT‐qPCR, following the manufacturer's instructions.

### 2.5 CNC analysis

Coding and non‐coding co‐expression analysis is based on the normalized signal intensity of the individual genes to identify any interactions between mRNAs and circRNAs. On the basis of correlation analysis, we constructed a CNC network between DE mRNAs and related circRNAs. The circRNA‐mRNA pairs with Pearson's correlation coefficient ≥.95 were identified and chosen to construct a network using Cytoscape software (version 2.8.3; Cytoscape Consortium). To completely understand the CNC network, GO and KEGG analyses were performed for targeted mRNAs.

### 2.6 Construction of circRNA‐miRNA‐mRNA ceRNA regulatory network

CircRNAs are known to adversely regulate miRNA expression and substantially contribute to the ceRNA network. First, the circRNA/miRNA interaction was predicted using Arraystar's home‐made miRNA target prediction software based on TargetScan^12^ and miRanda.^13^ Next, miRanda^13^ and TargetScan^12^ databases were used to identify miRNA‐mRNA pairs. Finally, circRNA‐miRNA‐mRNA networks were constructed. For the complete understanding of ceRNA effects, GO and KEGG analyses were performed for targeted mRNAs.

### 2.7 Statistical analysis

Quantile normalization of raw data and subsequent data processing were performed with the R software limma package. Statistical significance of difference was conveniently estimated by a *t* test. CircRNAs or mRNAs with fold changes ≥1.5 and *P* ≤ .05 were considered to exhibit significant differential expression. Quantitative data were presented as mean ± standard error of the mean. Pearson's correlation analysis was used to detect any relationship between circRNAs and mRNAs. Statistical analysis was performed with GraphPad Prism 6 (GraphPad Software).

## 3 RESULTS

### 3.1 Differential expression profiles of circRNAs and mRNAs between LPS and control groups

**Figure 1:**
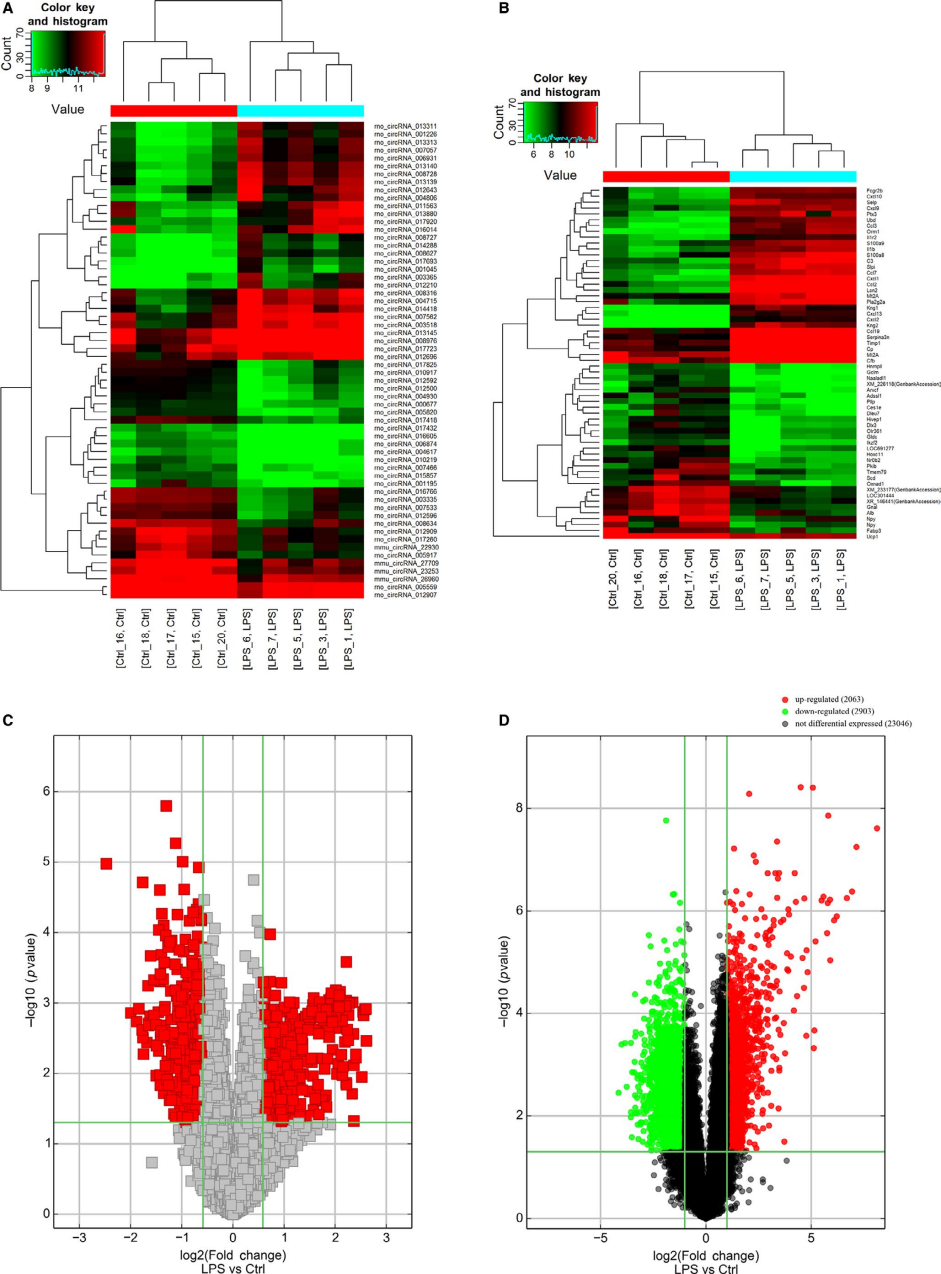
Changes in the expression profiles of circRNAs and mRNAs. Heat maps showing the expression profiles of (A) circRNAs and (B) mRNAs. Volcano plots presenting differences in the expression of (C) circRNAs and (D) mRNAs between LPS and Ctrl groups. Values plotted on the *x*‐ and *y*‐axes represent the averaged normalized signal values of each group (log2‐scaled). Ctrl, control

We established a sepsis model through an intraperitoneal injection of LPS, as evident from the decrease in the MAP level to 30% or below. There was no significant change in blood pressure in rats injected with saline. Aside from the significant decrease in MAP level, signs of shock such as lassitude, tachycardia and a sharp drop in body temperature were observed in all rats. Total RNA was extracted from the aorta tissue and used for circRNA and mRNA high‐throughput sequencing. In comparison with the control group, septic rats had 373 up‐regulated and 428 down‐regulated circRNAs in the aorta tissue after applying filtering conditions (fold change ≥ 1.5 and *P* < .05). The results of mRNA sequencing revealed 2063 up‐regulated and 2903 down‐regulated mRNAs in the aorta tissue of septic rats. Heat maps showed the expression profiles of the top 50 distinct circRNAs (Figure 1A) and mRNAs (Figure 1B) between LPS and control groups in terms of fold change, while volcano plots revealed the variations in circRNA expression patterns between the two groups (Figure 1C,D). Table S1 shows the top 20 DE circRNAs, while the top 20 DE mRNAs are shown in Table S2. The data supporting the findings of this study are openly available in the GenBank database under the accession numbers GSE147772 and GSE147774.

### 3.2 GO and KEGG pathway analyses of DE mRNAs

**Figure 2:**
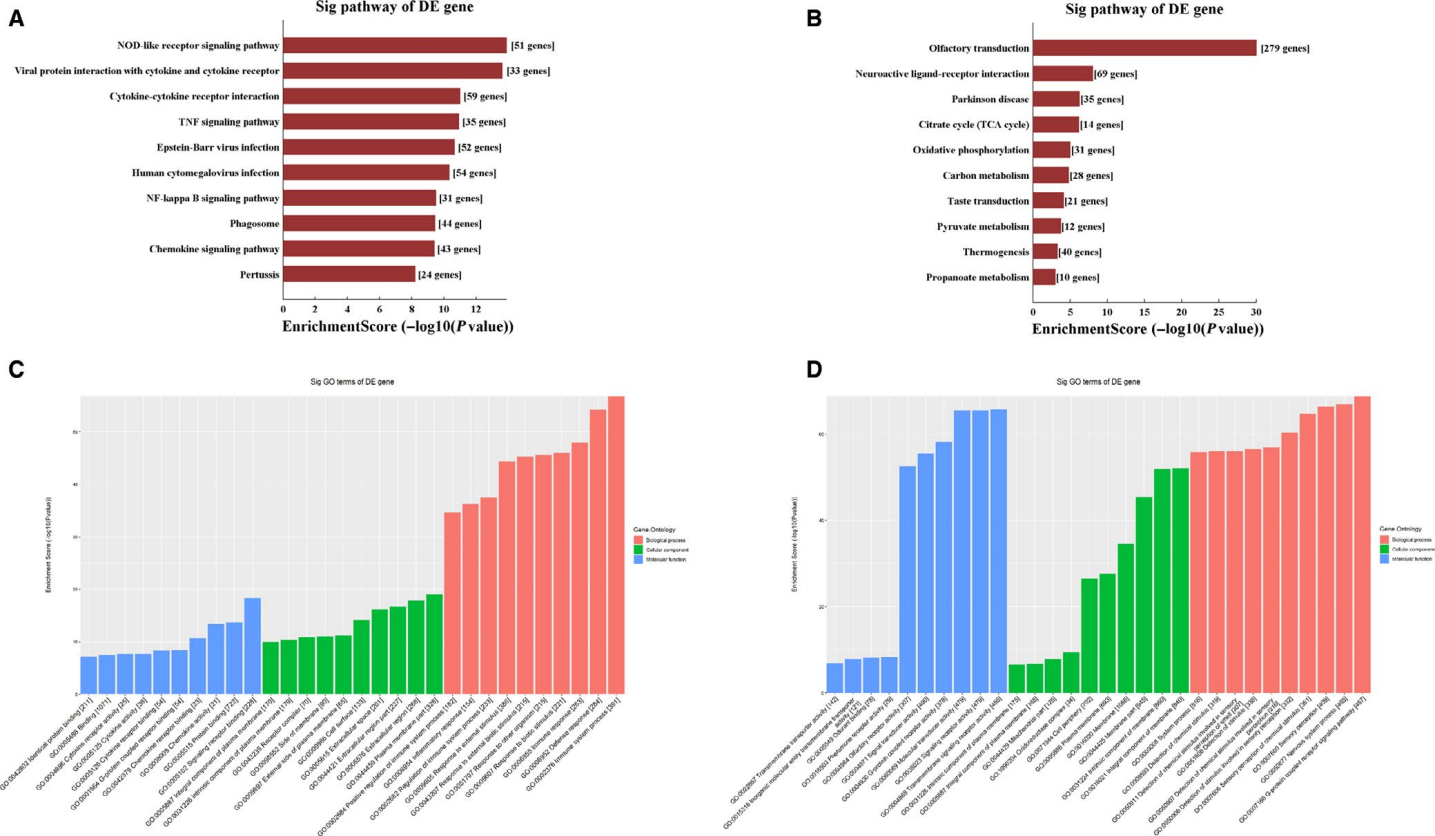
Gene Ontology pathway analysis of differentially expressed mRNAs. Kyoto Encyclopedia of Genes and Genomes pathway enrichment for up‐regulated (A) and down‐regulated (B) mRNAs. Gene Ontology enrichment analysis for up‐regulated (C) and down‐regulated (D) mRNAs

According to KEGG analysis, the up‐regulated DE mRNAs were mostly enriched in the NOD‐like receptor signalling pathway (Figure 2A), while the most significantly enriched pathway for the down‐regulated DE mRNAs was olfactory transduction (Figure 2B).

The top 10 results with the most significant *P*‐values in BP, CC and MF are shown as a bar plot. GO term analysis of up‐regulated DE mRNAs revealed the most enriched GO terms to be immune system process (BP), signalling receptor binding (MF) and plasma membrane part (CC) (Figure 2C), while the most enriched GO terms for down‐regulated DE mRNAs were G protein‐coupled receptor signalling pathway (BP), transmembrane signalling receptor activity (MF) and integral component of membrane (CC) (Figure 2D).

### 3.3 Validation of circRNA and mRNA microarray results using RT‐qPCR

**Figure 3:**
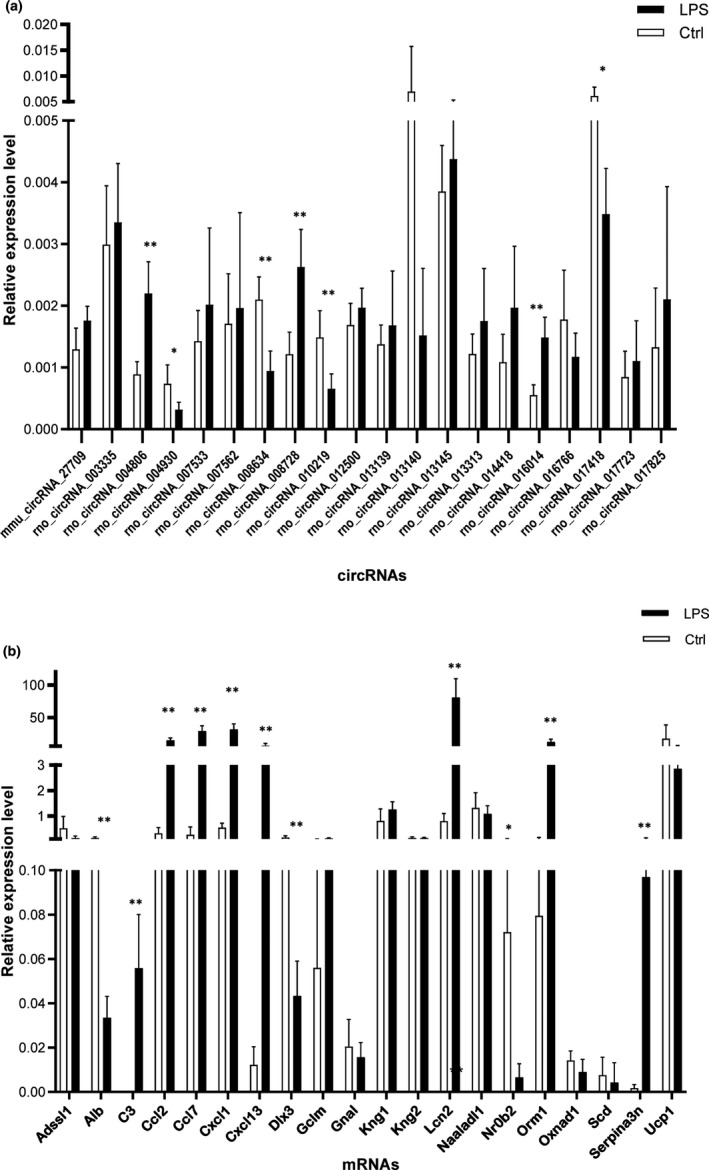
Validation of circRNA and mRNA expression. A, The relative expression levels of selected circRNAs, as detected by RT‐qPCR. B, The relative expression levels of selected mRNAs, as detected by RT‐qPCR. β‐actin was used as a housekeeping gene for normalizing changes in specific gene expression. *P < .05 and **P < .01 vs control group, n = 5 per group. RT‐PCR, real‐time quantitative polymerase chain reaction

We performed RT‐qPCR to validate microarray results and quantify the expression of the 20 selected DE circRNAs and 20 DE mRNAs in the aorta tissue. Of these, rno_circRNA_004806, rno_circRNA_004930, rno_circRNA_008634, rno_circRNA_008728, rno_circRNA_010219, rno_circRNA_016014 and rno_circRNA_017418 expression levels were consistent with the results of circRNA microarray analysis (Figure 3A). The primers used are listed in Table S3. In addition, the results for *Alb*, *C3*, *Ccl2*, *Ccl7*, *Cxcl1*, *Cxcl13*, *Dlx3*, *Lcn2*, *Nr0b2*, *Orm1* and *Serpina3n* were consistent with those of mRNA microarray analysis (Figure 3B). The corresponding primers are shown in Table S4.

### 3.4 CNC network analysis

**Figure 4:**
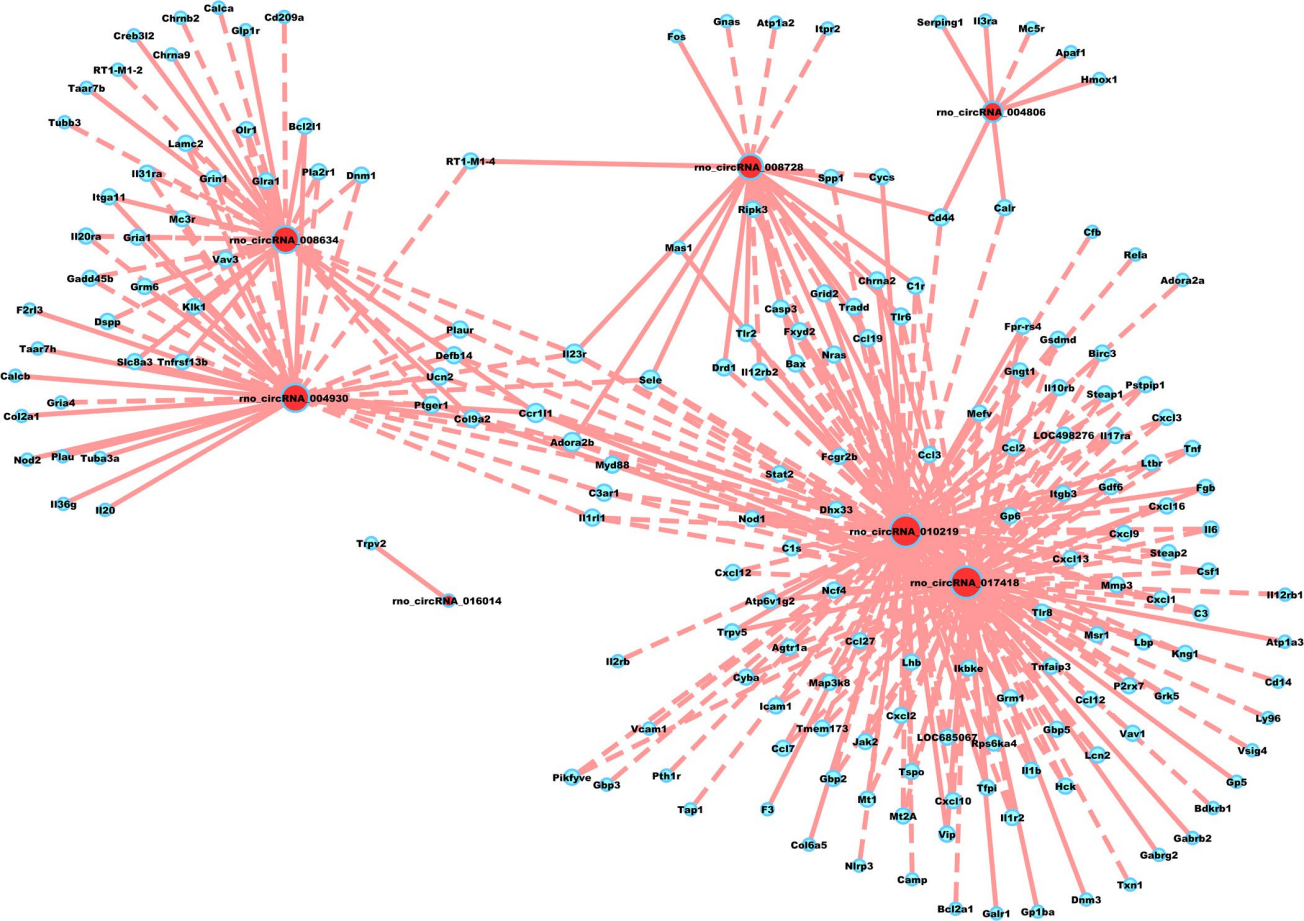
Coding and non‐coding co‐expression network. Red nodes are circRNAs; blue nodes are mRNAs. Positive correlation is indicated as a solid line, and negative correlation, as a dashed line

**Figure 5:**
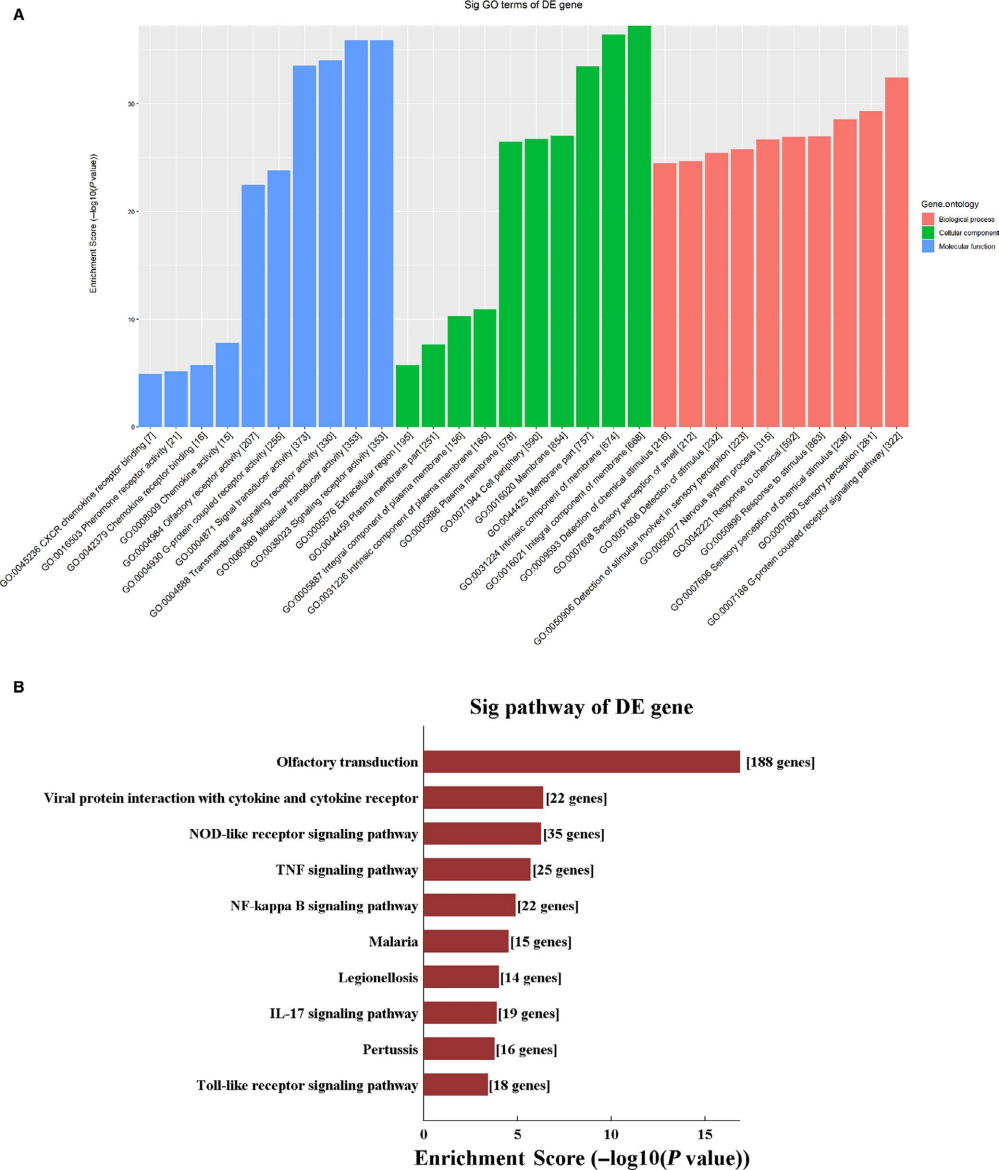
Gene Ontology pathway analysis of coding and non‐coding co‐expression network. A, Gene Ontology and (B) Kyoto Encyclopedia of Genes and Genomes pathway enrichment based on CNC analysis results. CNC: coding and non‐coding co‐expression

We carried out CNC analysis using the seven validated DE circRNAs in combination with the DE mRNAs obtained from microarray results of rat vascular whole‐genome expression profiling. The predicted target genes were used to conduct KEGG and GO analyses. This allowed us to screen the pathways related to or potentially associated with the research background. The enriched genes were screened and summarized to construct a CNC network (Figure 4). The top two enriched BPs were G protein‐coupled receptor signalling pathway and sensory perception, while the top two enriched CCs were integral component of membrane and intrinsic component of membrane. In addition, the top two enriched MFs were signalling receptor activity and molecular transducer activity (Figure 5A). KEGG pathway analysis revealed the top three pathways, namely, olfactory transduction, viral protein interaction with cytokine and cytokine receptor, and NOD‐like receptor signalling pathway (Figure 5B).

### 3.5 Construction of ceRNA networks

**Figure 6:**
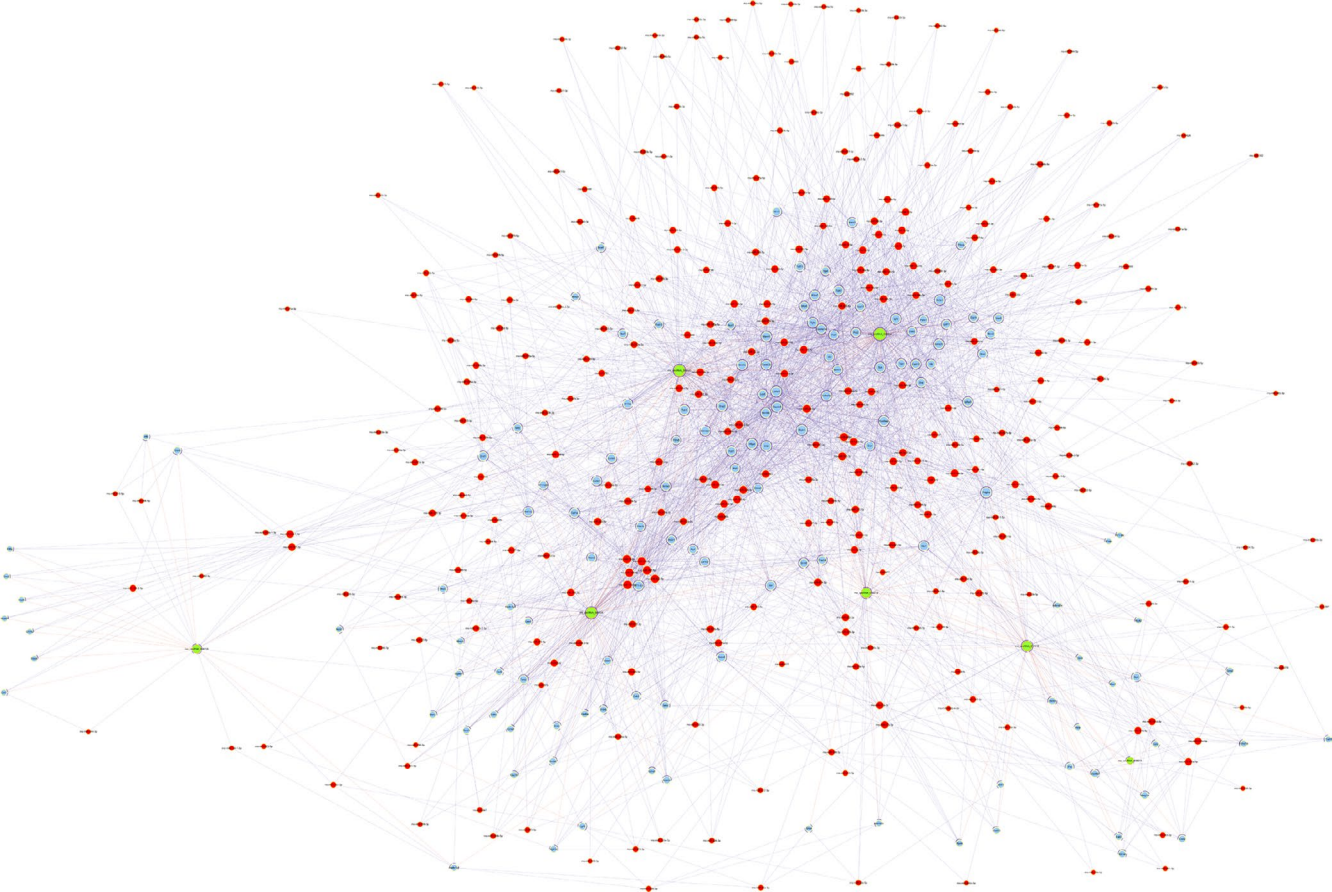
Competing endogenous RNA network. Red circles represent miRNAs, blue circles represent mRNAs, and green circles represent circRNAs

**Figure 7:**
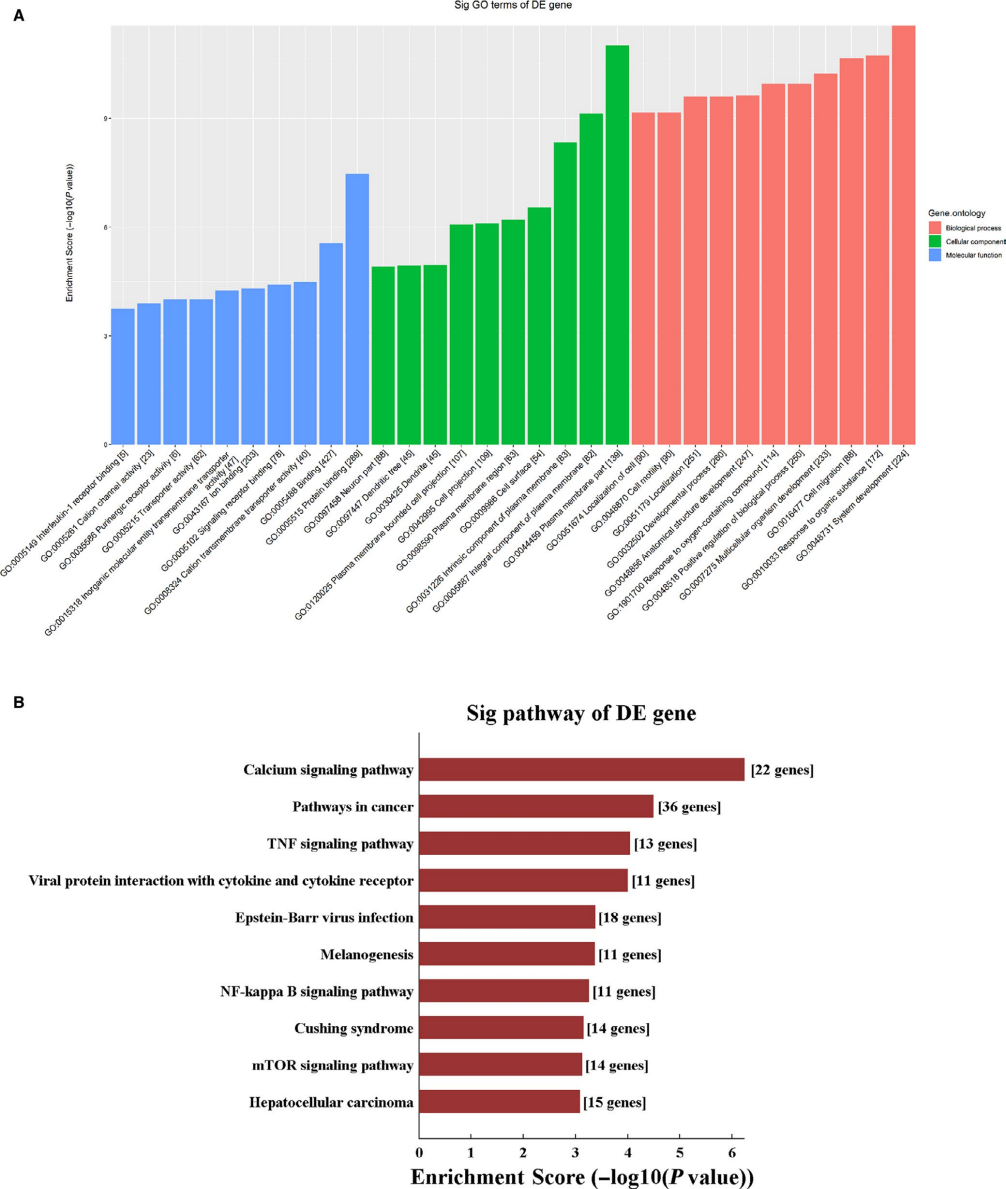
Gene Ontology pathway analysis of competing endogenous RNA network. A, Gene Ontology and (B) Kyoto Encyclopedia of Genes and Genomes pathway enrichment based on ceRNA analysis results

We performed ceRNA analysis with the seven validated DE circRNAs and DE mRNAs. The number of predicted miRNA‐ID was confined within 1000. The predicted target genes were used to conduct KEGG and GO analyses, which revealed the pathways related to the research background. The enriched genes were used to construct the ceRNA network (Figure 6). The top two enriched BPs were system development and response to organic substance, while plasma membrane part and integral component of plasma membrane served as the top two enriched CCs. In addition, the top two enriched MFs were protein binding and binding (Figure 7A). KEGG pathway analysis revealed the top three riched pathways, namely, calcium signalling pathway, pathways in cancer, and tumour necrosis factor (TNF) signalling pathway (Figure 7B).

## 4. DISCUSSION

4

Given the important functions of circRNAs in various biological processes, these molecules have attracted attention among researchers.^3^, ^14^ RNA sequencing is a powerful tool that can not only decipher the non‐annotated transcriptional activity but also reveal the differential expression profile of RNAs underlying specific phenotypic difference. To explore the potential role of circRNAs in sepsis, we established a rat model of sepsis through intraperitoneal injection of LPS, and extracted the aorta tissue for high‐throughput sequencing of circRNAs and mRNAs. Our results show that 373 and 428 circRNAs were up‐regulated and down‐regulated and 2063 and 2903 mRNAs were up‐regulated and down‐regulated, respectively, in septic aorta tissues as compared with those in control tissues.

We performed GO and KEGG analyses of DE mRNAs. KEGG analysis showed that the up‐regulated mRNAs were enriched in the pathways related to inflammatory response, such as cytokine‐cytokine receptor interaction, TNF signalling pathway, nuclear factor kappa‐B signalling pathway and chemokine signalling pathway. Thus, inflammatory response may be strongly activated in LPS‐stimulated vascular tissues, leading to the release of several inflammatory factors that contribute to a cytokine storm.^15^ The most common feature of cardiovascular dysfunction in patients with sepsis is arterial hypotension, mainly contributed. According to published reports, the key phenomena involved in the pathophysiology of shock are hypovolaemia and decreased vascular tension.^16^ LPS stimulation leads to the activation of vascular endothelial cells that release several inflammatory mediators (TNF‐α, interleukin [IL]‐1β), chemokines (monocyte/macrophage chemotactic protein‐1 [MCP‐1] and IL‐8), adhesion molecules (intercellular cell adhesion molecule‐1 [ICAM‐1] and vascular cell adhesion molecule‐1 [VCAM‐1]), coagulation factor (tissue factor and plasminogen activator inhibitor‐1 [PAI‐1]) and damage thrombomodulin (TM).^17^ LPS‐mediated excessive activation of macrophages may also lead to the overproduction of pro‐inflammatory cytokines, which, in turn, produce a cytokine storm.^15^ We observed up‐regulation in mRNA molecules in association with various inflammatory responses, consistent with the observations of previous study reports. The released inflammatory factors, chemokines, adhesion molecules and coagulation factors further promote vascular endothelial damage. Endothelial cell damage may affect vascular functions in several ways involving barrier function and vascular regulation.^18^ Any impairment in vascular barrier function or increase in permeability may cause plasma extravasation, tissue oedema and hypovolaemia. Upon stimulation with LPS, macrophages can attract classically activated macrophages that produce nitric oxide (NO). An increase in the level of NO may decrease the vascular tone. The damage caused to endothelial cells may increase the level of NO, and the down‐regulation of endothelial‐related antioxidant defence system would result in the up‐regulation in the generation of reactive oxygen species, exposure of adhesion molecules and activation of tissue factors, which are related to the decrease in vascular tension. Therefore, LPS can cause hypovolaemia by damaging endothelial cells, increasing vascular permeability and releasing NO to reduce vascular tone, ultimately leading to hypotension shock. Circulatory failure is a key factor underlying the progression of sepsis into organ failure. As endothelial dysfunction plays an important role in sepsis, it is imperative to control inflammatory response, reduce release of inflammatory factors and protect the vascular system in an attempt to improve the survival rate of septic patients.

The down‐regulated mRNAs were significantly enriched in the pathways related to energy metabolism such as citrate cycle, oxidative phosphorylation and pyruvate metabolism. GO analysis of the down‐regulated mRNAs suggested the significant enrichment of many CCs related to energy generation, such as oxidoreductase complex, mitochondrial part, respiratory chain, mitochondrial inner membrane and respiratory chain complex. Oxidative phosphorylation in mitochondria is destroyed in an inflammatory environment, resulting in the shortage of ATP supply.^19^, ^20^, ^21^, ^22^ Sepsis affects mitochondrial respiration and specific electron transport chain complexes in animal models and septic patients.^23^ The sepsis‐induced loss of contractility is not accompanied with cardiomyocyte death but instead is related to the decreased activity of mitochondrial cytochrome c oxidase. Improvement in the activity of cytochrome c oxidase by exogenous cytochrome c supplementation may result in the restoration of cardiac contractility.^24^ In skeletal muscle biopsies from patients with severe sepsis, the complex I and IV activities of mitochondrial electron transport chain were significantly reduced.^25^, ^26^ Non‐survivors had significantly decreased complex I and IV activities along with reduced ATP levels. The cardiovascular system is essential to maintain adequate organ perfusion. Therefore, cardiovascular dysfunction seriously affects the progression of sepsis. Severe circulatory abnormalities and the presence of metabolic and cellular disorders are associated with high mortality in some patients. In the present study, high‐throughput sequencing of mRNAs in the aorta tissue revealed the down‐regulated expression of many molecules involved in energy generation. We suspect that the expression of many molecules involved in energy generation in the aorta tissue is down‐regulated upon LPS stimulation; this may decrease ATP production and consequently lead to decreased blood pressure. Therefore, the choice of drugs that improve energy metabolism may be helpful to treat sepsis.

Studies have shown that circRNAs play different roles in sepsis‐induced organ dysfunction. However, most functions of circRNAs are unclear. The construction of CNC and ceRNA networks is an effective strategy to predict the functions of circRNAs. After RT‐qPCR verification, we selected seven most distinct circRNAs for CNC and ceRNA analyses to investigate the downstream regulatory genes and their pathways. The seven circRNAs were rno_circRNA_004806, rno_circRNA_004930, rno_circRNA_008634, rno_circRNA_008728, rno_circRNA_010219, rno_circRNA_016014 and rno_circRNA_017418.

We performed GO and KEGG analyses on the downstream target genes predicted by CNC. These signalling pathways related to inflammatory reactions, including viral protein interaction with cytokine and cytokine receptor, TNF signalling pathway, nuclear factor kappa‐B signalling pathway and IL‐17 signalling pathway, were significantly enriched. Although their functions are still unclear, circRNAs are thought to be involved in the regulation of inflammatory response.^27^ In a mouse model of traumatic brain injury, 191 DE circRNAs were found in the cortex; enrichment analysis showed that these miRNAs were mainly associated with inflammation.^28^ In nucleus pulposus cells, TNF‐α was shown to elevate circ‐4099 expression by up‐regulating glucose‐related protein 78 (GRP78) levels. Circ‐4099 enhances the synthesis of the extracellular matrix by sponging miR‐616‐5p.^29^ Controlling circ‐4099 activity during disc disease may, thus, delay or reverse the process of disc degeneration. In stroke models, circ‐DLGAP4 sponges the pro‐inflammatory gene miR‐143, suggesting the involvement of circ‐DLGAP4 in inflammatory response.^30^, ^31^, ^32^ The circRNA RasGEF1B acts as a positive regulator of ICAM‐1 of the Toll‐like receptor 4 (TLR4)/LPS pathway, an essential signalling pathway in inflammatory response.^33^ In addition, circRNA‐9119 is known to sponge both miR‐26a and miR‐136, which, in turn, regulate TLR3 and inducible gene I, the two essential molecules in orchitis.^34^ We performed CNC analysis of seven distinct circRNAs and found that their downstream target genes were significantly associated with inflammatory response; thus, circRNAs may be involved in LPS‐induced vascular inflammation. CircRNAs may regulate the level of pro‐inflammatory cytokines and stimulate inflammatory and immune responses, modulate the vascular function during sepsis and influence sepsis progression. How circRNAs regulate downstream mRNA molecules is questionable and warrants further research.

Although the function of circRNAs is uncertain, their “miRNA sponge effect” has been well documented. The binding of circRNAs to their target miRNAs results in the down‐regulation of the expression of target mRNAs; here, circRNAs act as sponges, inhibit mRNA transcription and affect the pathophysiological processes of various diseases. We constructed a circRNA‐miRNA‐mRNA regulatory network based on biological prediction, and performed GO and KEGG analyses of the predicted downstream target genes. KEGG analysis showed that the calcium signalling pathway was the most significantly enriched pathway. In general, intracellular calcium binds to calmodulin, activates calmodulin kinases, and further regulates inflammation and immunity. Previous studies have shown that sepsis increases calcium leakage from the sarcoplasmic reticulum of cardiomyocytes.^35^ Treatment of isolated adult mouse cardiomyocytes with LPS results in an increase in the total cellular reactive oxygen species and mitochondrial superoxide levels, leading to calcium overload and cardiomyocyte injury.^36^ LPS can induce calcium influx in mouse macrophages and up‐regulate the production of mediators such as NO and IL‐10.^37^ LPS can also induce extracellular calcium influx in mouse peritoneal macrophages and inhibit calcium influx, consequently leading to a significant increase in IL‐12 level.^38^ Our results suggest that aberrantly expressed circRNAs show extensive interactions with miRNAs, which play regulatory functions in calcium signalling pathway. These data indicate that circRNA‐related ceRNA networks may have key roles in the progression of vascular injury during sepsis. However, given that these results are based only on bioinformatic models, further in‐depth study is essential to verify the role of these seven circRNAs in vascular injury in sepsis.

Our high‐throughput sequencing results for mRNAs revealed the significant up‐regulation in the expression of proteins associated with inflammation, suggesting that the potential treatment of sepsis should first control the inflammatory storm and reduce the release of cytokines. This may greatly reduce mortality in septic patients. We also found that circRNAs regulate pro‐inflammatory cytokine levels and stimulate inflammatory and immune responses, providing a new direction to control inflammatory and immune responses. A potential drug that regulates one key circRNA may be useful to suppress the occurrence of inflammatory storm. In addition, we show for the first time that circRNAs regulate calcium signalling pathway, which may serve as a novel therapeutic target. Some calcium antagonists can be used to reduce the vascular damage in septicaemia and to improve organ perfusion. Our high‐throughput sequencing data of mRNA also showed the significant down‐regulation in the expression of proteins related to energy metabolism, suggesting that the rational use of drugs to improve energy metabolism may be helpful to reduce organ failure.

## 5 CONCLUSIONS

In this study, we comprehensively analysed the expression of circRNAs and mRNAs in the aorta tissue of an LPS‐induced sepsis rat model. Significantly, DE circRNAs and mRNAs were identified, and circRNA‐mRNA co‐expression and ceRNA networks were established to explore the regulatory relationship between coding genes and non‐coding genes. Comprehensive analysis showed that circRNAs played an important role in vascular injury in sepsis. As the role of circRNAs in septic vascular injury has been incompletely understood, this analysis provides valuable resources and information for future studies.

## CONFLICT OF INTEREST

The authors confirm that there are no conflicts of interest.

## AUTHOR CONTRIBUTION

**Mu‐Wen Nie:** Conceptualization (equal); Data curation (equal); Formal analysis (equal); Writing–original draft (equal). **Ye‐Chen Han:** Data curation (equal); Formal analysis (equal); Investigation (equal); Methodology (equal); Writing–review & editing (equal). **Zhu‐Jun Shen:** Conceptualization (equal); Formal analysis (equal); Investigation (equal); Methodology (equal); Software (equal); Writing–review & editing (equal). **Hong‐Zhi Xie:** Conceptualization (equal); Data curation (equal); Funding acquisition (equal); Project administration (equal); Resources (equal); Supervision (equal); Validation (equal); Visualization (equal); Writing–review & editing (equal).

## Supporting information

Table S1‐S4

## Data Availability

The data that support the findings of this study are openly available in the GenBank databases under accession number GSE147772 and GSE147774. Addresses are as follows: https://www.ncbi.nlm.nih.gov/geo/query/acc.cgi?acc=GSE147772 https://www.ncbi.nlm.nih.gov/geo/query/acc.cgi?acc=GSE147774
