# Supplementary material for: Identification of circRNA and mRNA expression profiles and functional networks of vascular tissue in lipopolysaccharide‐induced sepsis
Source: J Cell Mol Med. 2020 May 25;24(14):7915–27. doi: 10.1111/jcmm.15424 (PMC7348180; doi:10.1111/jcmm.15424)
Supplement: Supplementary file 1 — Table S1‐S4 [file JCMM-24-7915-s001.docx]

**Supplementary Material**

**Content**

**Table S1** The detailed information of the top ten up-regulated and top ten down-regulated circRNAs

**Table S2** The detailed information of the top ten up-regulated and top ten down-regulated mRNAs

**Table S3** Primers for circRNAs validated by qRT-PCR

**Table S4** Primers for mRNAs validated by qRT-PCR

**Table S1** The detailed information of the top ten up-regulated and top ten down-regulated circRNAs

| **circRNA** | **Fold change** | **Regulation** | **chrom** | **strand** | **circRNA_type** | **GeneSymbol** |
| --- | --- | --- | --- | --- | --- | --- |
| rno_circRNA_010219 | 5.6 | down | chr3 | - | exonic | Pak7 |
| mmu_circRNA_27709 | 4.0 | down | chr15 | + | sense overlapping | Kif13b |
| rno_circRNA_003335 | 3.7 | down | chr11 | + | sense overlapping | Mb21d2 |
| rno_circRNA_004930 | 3.6 | down | chr14 | - | sense overlapping | Nsun7 |
| rno_circRNA_016766 | 3.4 | down | chr8 | - | exonic | Tbc1d2b |
| rno_circRNA_017418 | 3.4 | down | chr9 | - | exonic | Erbb4 |
| rno_circRNA_012909 | 3.4 | down | chr5 | - | exonic | Kdm1a |
| rno_circRNA_005917 | 3.4 | down | chr16 | - | sense overlapping | 5S_rRNA |
| rno_circRNA_008634 | 3.2 | down | chr2 | + | sense overlapping | Ash1l |
| rno_circRNA_012500 | 3.2 | down | chr5 | + | sense overlapping | Pde4b |
| rno_circRNA_013145 | 6.1 | up | chr5 | - | sense overlapping | Asph |
| mmu_circRNA_21243 | 6.0 | up | chr13 | + | exonic | Tmco1 |
| rno_circRNA_008728 | 6.0 | up | chr2 | - | exonic | Man1a2 |
| rno_circRNA_013313 | 5.9 | up | chr5 | - | exonic | Gabbr2 |
| rno_circRNA_013139 | 5.8 | up | chr5 | - | exonic | Asph |
| mmu_circRNA_007951 | 5.7 | up | chr19 | + | intergenic |  |
| rno_circRNA_004806 | 5.3 | up | chr14 | + | exonic | Evi5 |
| rno_circRNA_014418 | 5.3 | up | chr6 | + | sense overlapping | Dgkb |
| rno_circRNA_016014 | 5.1 | up | chr8 | - | exonic | Zfp426 |
| rno_circRNA_017723 | 5.1 | up | chrX | - | exonic | Smarca1 |

**Table S2** The detailed information of the top ten up-regulated and top ten down-regulated mRNAs

| **ProbeName** | **P-value** | **Fold Change** | **Regulation** | **GeneSymbol** |
| --- | --- | --- | --- | --- |
| A_42_P638620 | 2.45637E-08 | 278.5 | up | Lcn2 |
| A_42_P473398 | 5.68236E-08 | 141.6 | up | Cxcl1 |
| A_44_P348781 | 4.19483E-07 | 122.6 | up | Kng2 |
| A_42_P695401 | 5.65365E-07 | 103.3 | up | Ccl2 |
| A_44_P1022002 | 1.27755E-06 | 74.0 | up | Ccl7 |
| A_64_P137063 | 1.53403E-06 | 67.8 | up | Kng1 |
| A_44_P1023538 | 9.19784E-06 | 59.8 | up | C3 |
| A_44_P146845 | 6.05419E-07 | 59.4 | up | Orm1 |
| A_44_P1000653 | 1.39841E-08 | 56.0 | up | Serpina3n |
| A_64_P013437 | 7.01707E-07 | 55.0 | up | Cxcl13 |
| A_44_P603503 | 0.003540242 | 17.6509125 | down | Oxnad1 |
| A_64_P013122 | 0.000379061 | 14.3153195 | down | Gnal |
| A_44_P1017367 | 0.002693932 | 13.5333716 | down | Alb |
| A_64_P049223 | 0.010601019 | 11.7817856 | down | Olr361 |
| A_44_P1011898 | 0.001405221 | 11.2960469 | down | Gclm |
| A_64_P113621 | 0.008802087 | 10.3196559 | down | Arvcf |
| A_42_P614984 | 4.25552E-05 | 10.2829585 | down | Ucp1 |
| A_64_P135713 | 0.003970728 | 10.2691443 | down | Dlx3 |
| A_43_P13041 | 0.000281404 | 10.2258576 | down | Nr0b2 |
| A_64_P085530 | 0.006910931 | 10.1987362 | down | Scd |

**Table S3** Primers for circRNAs validated by qRT-PCR

| Gene | Forward and Reverse primer sequence |
| --- | --- |
| GAPDH(RAT) | F:5’ GCTCTCTGCTCCTCCCTGTTCTA3'  R:5’ TGGTAACCAGGCGTCCGATA3’ |
| rno_circRNA_013145 | F:5’ GCAGACGGTGTTGATGAGGC 3’  R:5’ CTCTCCGCCCATTCTTGTGTC 3’ |
| rno_circRNA_008728 | F:5’ AGTTAACATTCGCTTTATTGG 3’  R:5’ AGACGCTCTTCTTCTTCCTTC 3’ |
| rno_circRNA_013313 | F:5’ GATTCTCACCGTGGGCTAC 3’  R:5’ CTTCACCTCTCTGCTGTCTTCT 3’ |
| rno_circRNA_013139 | F:5’ TGGCTACACAGAGCTAGTGAAGT 3’  R:5’ TTCGATTTTACCCCTTTTCC 3’ |
| rno_circRNA_004806 | F:5’ CAGAGATGAGAGAGATGAAGCA 3’  R:5’ TCACCCATGAGAGAGTAGAACT 3’ |
| rno_circRNA_014418 | F:5’ TTAGGAGGTGCCATTACTCGT 3’  R:5’ ATGCATGCTTGGTATATTCAAA 3’ |
| rno_circRNA_016014 | F:5’ GAAGAGTTTAGTACAGGGCAGAA 3’  R:5’ AGGCAATCAACCAACATCATT 3’ |
| rno_circRNA_017723 | F:5’ GAAACGGGCAACTAAAACTCC 3’  R:5’ TTCTCTTCATATTCTGGGTCCAT 3’ |
| rno_circRNA_013140 | F:5’ CAAGATACGGCAAAGCACAGT 3’  R:5’ TTTCTGCAGCGTCCAGCTC 3’ |
| rno_circRNA_010219 | F:5’ ACAACTCCTACACCTACCCTCG 3’  R:5’ GGATTTATTTCCTCTGACGATT 3’ |
| mmu_circRNA_27709 | F:5’ CTCCAGCTCTCTCTGTTTCGC 3’  R:5’ CATCCTTGCCAGTGCCTCT 3’ |
| rno_circRNA_003335 | F:5’ GACCGAAATGGAGAGCAAAG 3’  R:5’ CCAATGAGACAAAGAGCCTTC 3’ |
| rno_circRNA_004930 | F:5’ GCCCAGCACTTAAACTACAACC 3’  R:5’ CTGGCAGAATATGGTAAATCGA 3’ |
| rno_circRNA_016766 | F:5’ ACACCAATGAAGAGTGGGAACT 3’  R:5’ GGTGCTGGGAAATTATATCTTT 3’ |
| rno_circRNA_017418 | F:5’ GCCTGAAGGTGAAACTGTGAA 3’  R:5’ ATAGCATTGTAAGGGTCAATCC 3’ |
| rno_circRNA_008634 | F:5’ CGGGAGAGCATGAGTATGG 3’  R:5’ CAGCAATTCCTGAGAAACAAC 3’ |
| rno_circRNA_012500 | F:5’ CACGCTACCTTTGACGACACTT 3’  R:5’ TGCTGACTGCAGACTAGACCAC 3’ |
| rno_circRNA_007562 | F:5’ GCTTGGAGGAGCAGGAGTTC 3’  R:5’ AACGTCCCGAGAATGGAGAC 3’ |
| rno_circRNA_017825 | F:5’ AGGAGCAAAGCATTCCCAG 3’  R:5’ ACCAACATGTCCACAGAAGTCT 3’ |
| rno_circRNA_007533 | F:5’CAGTCTGAAAAAAGATGAAGTGTG 3’  R:5’ CACTGATCTACCGTATTTGCTG 3’ |

**Table S4** Primers for mRNAs validated by qRT-PCR

| Gene | Forward and Reverse primer sequence |
| --- | --- |
| GAPDH(RAT) | F:5’ GCTCTCTGCTCCTCCCTGTTCTA3'  R:5’ TGGTAACCAGGCGTCCGATA3’ |
| Lcn2 | F:5’ CGTCACTTCCATCCTCGTCA 3’  R:5’ CTGGTCGTAGTCAGTGTCGG 3’ |
| Cxcl1 | F:5’ GTGTTTTGTGTTAGGGTGAGG 3’  R:5’ GACGAGAAGGAGCATTGGTTA 3’ |
| Kng2 | F:5’ CGTGGAGCAGAGTCCTGTTT 3’  R:5’ ATTCCTGCAGTCACCCCTTG 3’ |
| Ccl2 | F:5’ GCTACTCATTCACTGGCAAGA 3’  R:5’ CTTATTGGGGTCAGCACAGAT 3’ |
| Ccl7 | F:5’ CGCTTCTGTGTGTGCTGCTCA 3’  R:5’ GCCTCCTCAACCCACTTCTG 3’ |
| Kng1 | F:5’ TTTTTGGCAAAACAATTCCTCA 3’  R:5’ GCAGTCCATTTCCTGGGCA 3’ |
| C3 | F:5’ TGGACCATAGAAGAGTTGAA 3’  R:5’ CACAGAGTAGGGCAGTCG 3’ |
| Orm1 | F:5’ ACTTCGGGAGTTTCAGACCAC 3’  R:5’ CTTCAGCACTATCAAATGGGC 3’ |
| Serpina3n | F:5’ ATCAGGAGTCGGCAATCACA 3’  R:5’ AAGTCACAAGGCGGGTCATC 3’ |
| Cxcl13 | F:5’ AAGTTATACGCCCTGGGAATG 3’  R:5’ GCCGTGTTTGTAGAGGGAAGTT 3’ |
| Oxnad1 | F:5’ CAGCACTCTGCGTTCCCTTAC 3’  R:5’ AAACCCACCAACCACTGAAACT 3’ |
| Gnal | F:5’TCCCAAAGTTACAAGAGCCAAGTT 3’  R:5’GGTGAAGTGAGGGTAGCAGTAATGT 3’ |
| Alb | F:5’ AGCTGTCCGTCAGAGGATGA 3’  R:5’ AGCACTGGCTTATCACAGCA 3’ |
| Gclm | F:5’ CGCCTGCGGAAAAAGTGTC 3’  R:5’ CCACTGCATGGGACATGGTA 3’ |
| Ucp1 | F:5’ GCCTAGCAGACATCATCACCT 3’  R:5’ CCAGCCGAGATCTTGCTTCC 3’ |
| Dlx3 | F:5’ TTATGTGACCCTGTTCCTCCTG 3’  R:5’ CTGACTTCATTCCTGTCCCTTT 3’ |
| Nr0b2 | F:5’ GGCACTATCCTCTTCAACCCA 3’  R:5’ GGAAGCCATGAGGAGGATTCG 3’ |
| Scd | F:5’ ACATCCGTCCTGAAATGAGA 3’  R:5’ AGGGCACTGATAAGGTAGTAAA 3’ |
| Naaladl1 | F:5’ CAATTCCCACCCAGCCTATC 3’  R:5’ CCGGTCCCAGCTTGTACTCA 3’ |
| Adssl1 | F:5’ TGACCACGGGCAGGAAGA 3’  R:5’ GTTAGCAGGGAAGTAGGGGAT 3’ |
